# How and Why Chromosome Inversions Evolve

**DOI:** 10.1371/journal.pbio.1000501

**Published:** 2010-09-28

**Authors:** Mark Kirkpatrick

**Affiliations:** Section of Integrative Biology, University of Texas, Austin, Texas, United States of America

## Abstract

Chromosome inversions are a major engine of genome evolution. New genomic and ecological data are beginning to reveal the evolutionary forces that drive the evolution of inversions.

Alfred Sturtevant, who invented genetic mapping while still an undergraduate, published the first evidence of a chromosomal inversion in 1921 [1]. He suggested then, and later proved, that they have a dramatic effect on transmission: when heterozygous, inversions suppress recombination. Over the next half century, inspired largely by Dobzhansky and his coworkers, much of empirical population genetics devoted itself to studying the abundant polymorphisms within and fixed differences of inversions between species of *Drosophila* [2]. Starting in the 1970s, this rich literature largely sank from view with the rise of biochemical and then molecular genetics. But inversions are ascendant again. Comparative genomics is now revealing that chromosomes are far more structurally fluid than even Dobzhansky dared to suppose. Where classic cytogenetics identified only nine inversions that distinguish humans and chimpanzees, comparison of their genomic sequences reveals on the order of 1,500 [3] (Figure 1). Despite the importance of inversions as a major mechanism for reorganizing the genome, we are still struggling to understand how and why they evolve almost a century after Sturtevant's discovery.

**Figure 1:**
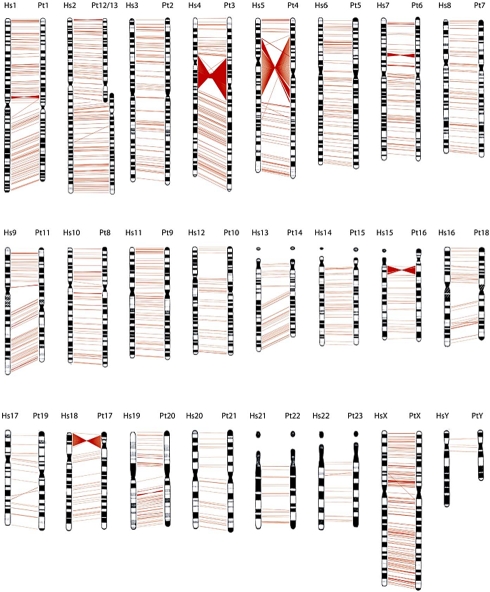
Chromosome inversions that distinguish humans and chimpanzees inferred from a comparison of their genomic sequences [3]. The human chromosome is shown on the left and its chimpanzee homologue on the right for the autosomes and the two sex chromosomes (X and Y). Each red line corresponds to an inversion, with larger inversions (>100 kb) represented by multiple lines.

An inversion occurs when a chromosome breaks at two points and the segment bounded by the breakpoints is reinserted in the reversed orientation. Several molecular mechanisms can mediate this event [4]. Box 1 gives an overview of some basic properties of inversions and the ways that they are detected.

**Box 1. What are chromosome inversions?**

Inversions are a diverse class of chromsomal mutation. The majority are small (<1KB) [3]. Others, for example the famous 3RP inversion of *Drosophila melanogaster*, are several megabases in size, include several percent of the entire genome and span hundreds or thousands of genes [10].

Inversions fall into two classes: *pericentric inversions* include a centromere, while *paracentric inversions* do not. With pericentric inversions, a single crossover event that occurs between the breakpoints of a heterozygote produces unbalanced gametes that carry deletions, insertions, and either zero or two centromeres. This can reduce fertility, making the inversions underdominant (lowered heterozygote fitness). Some pericentric inversions apparently escape fitness costs when heterozygous, however, perhaps because they somehow suppress recombination [33]. Although these may represent but a small fraction of all pericentric inversions that arise by mutation, they are likely to be greatly enriched among those that spread to fixation. There are large systematic differences between taxa in the frequency and severity of fitness effects. For example, heterozygotes for inversions seem to show decreased fertility in plants much more commonly than in animals [10].

By contrast, many of the paracentric inversions segregating in nature may not suffer from underdominance. This is likely a major reason why they are orders of magnitude more common than pericentric inversions, both as polymorphisms within and fixed differences between species [33].

Inversions were first seen in the giant salivary chromosomes of larval flies, and Diptera remains the group in which large inversions can be most easily detected. Chromosome staining techniques are able to visualize inversions in some other groups, including mammals, but with much lower resolution (and greater effort). The presence of an inversion is suggested when a certain cross consistently shows blocked recombination in part of the genome, but this observation requires genetic markers that have been mapped. Sequencing is a third way in which inversions are detected. The short reads that are characteristic of current high-throughput sequencing methods are well-suited to determine if an individual carries an inversion that has already been characterized by its breakpoints, but this technology is poor at prospecting for new inversions.

In many cases, there is virtually no difference in the genetic content of inverted and uninverted chromosomes—only the linear order of DNA bases is changed. This situation presents evolutionary biologists with an intriguing question: if an inverted chromosome has (almost) the same genetic information as an uninverted one, what could cause it to spread through a population? This primer begins with an overview of the evolutionary forces that act on inversions. It then discusses the importance of inversions to the evolution of sex chromosomes, speciation, and local adaptation. Finally, we will see how several of these themes are illuminated by an exciting study on an inversion in a plant that appears in this issue of *PLoS Biology*.

## Inversions and Recombination

**Figure 2:**
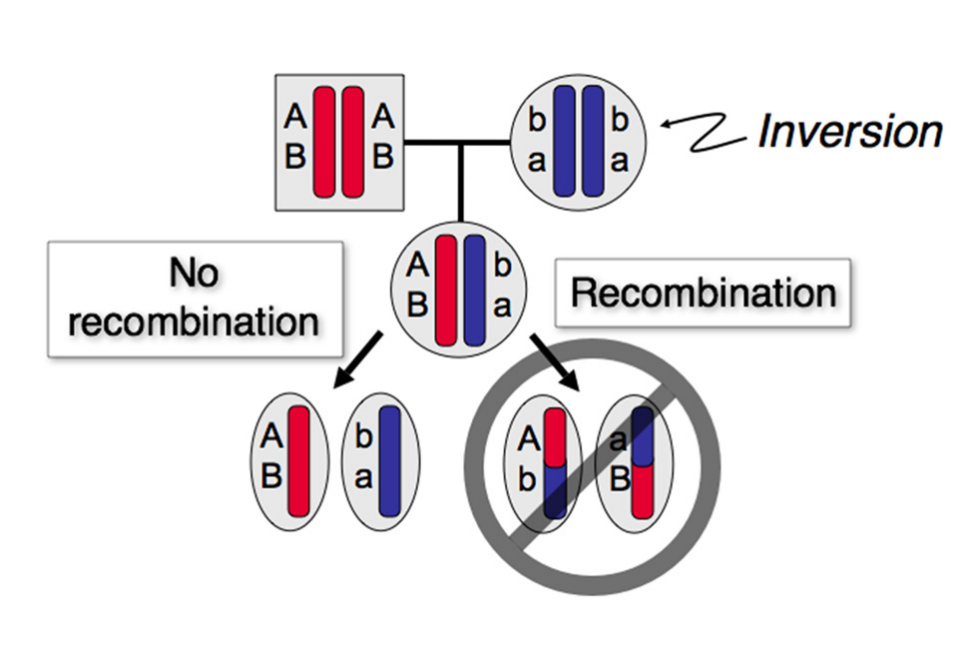
Schematic showing the suppression of recombination in an inversion heterozygote. Two loci segregate for the alleles (A, a) and (B, b). An individual that is heterozygous at both loci and for the inversion does not produce the recombinant gametes A/b and a/B.

A key evolutionary effect of inversions is that they suppress recombination as heterozygotes (Figure 2). Suppression follows from the loss of unbalanced gametes that result from recombination (Box 1), the failure of inverted regions to synapse in heterozygotes, and probably other mechanisms not yet understood. Large inversions show very low (but still positive) rates of recombination as heterozygotes, which results from double cross-overs and gene conversion, but the rates are orders of magnitude smaller than those in homozygotes [5]. On timescales of 10^5^ generations and longer, however, even this very limited recombination supplements mutation as an important source of genetic variation within inversions, which originate from a single chromosome and therefore have no variation whatsoever when they first appear.

One way to visualize the evolutionary properties of inversions is by an analogy. Consider the parallel between the populations of inverted and uninverted chromosomes, on the one hand, and a pair of coexisting biological species on the other. Within each of the two species, the normal rules of Mendelian inheritance apply. Between them, though, there is little genetic exchange, save for an occasional hybridization event (like the rare recombination between inverted and uninverted chromosomes). Ecological competition between the two species (like the fitness differences between the two chromosome forms) can either result in coexistence of the two species (like a stable polymorphism) or the replacement of one species by the other (like fixation of the ancestral or inverted chromosomal form).

## How Do Inversions Evolve?

Like other types of mutations, inversions evolve under selection and random drift. Many inversions, particularly small ones in intergenic regions, are likely to evolve neutrally (by drift alone). Selection can result in three ways. Inversions can generate structural problems with meiosis, as with some pericentric inversions. Alternatively, a breakpoint can disrupt an open reading frame or alter gene expression. The consequences can be deleterious, as in some human genetic diseases [6], but in other cases could cause an adaptive mutation. Finally, selection can act on an inversion when it carries one or more selected alleles.

Many pericentric inversions are underdominant (see Box 1), which poses an evolutionary puzzle. An underdominant inversion is selected against, so long as it's rare. Closely related species often show fixed differences, however, which implies that an inversion must have nevertheless appeared and spread through one of the two lineages since their last common ancestor. Some researchers have invoked drift to resolve this riddle [7],[8]. One line of support for that hypothesis is the observation that annual plants show high rates of evolution for underdominant chromosomal rearrangements [9],[10]. Many annual plants have large demographic fluctuations and at least occasionally self-fertilize, both of which dramatically decrease the effective population size and so enhance the power of drift.

Conversely, inversions can also be overdominant (superior as heterozygotes) [2]. The genetic basis for overdominance seems not to have been determined for any inversion. In principle, it could result from the effects of the breakpoints. Alternatively, it could result from overdominance at a locus within the inversion if the one allele is fixed on the ancestral chromosome and the other allele is fixed on the inverted chromosome. A third hypothesis is “associative overdominance.” This occurs when an inversion happens to capture one or more deleterious recessive alleles, which a large inversion is likely to do [11]. If the inversion is otherwise selectively favored when rare, it can spread to the point where recessive homozygotes become frequent enough to offset the initial advantage. The result is a balanced polymorphism that has the same evolutionary properties as conventional overdominance.

Some inversions show meiotic drive: the gametes of heterozygotes carry the inversion more than 50% of the time. Meiotic drive systems often involve a pair of interacting loci that must be coinherited for the system to invade a population. An inversion can suppress the recombination that would otherwise disrupt the drive system, and it then hitchhikes along as the driving alleles spread [12].

One reason that evolutionary biologists are fascinated by inversions is that they are highly polymorphic in some species [2]. Polymorphic inversions do not seem to be ancient, at least in flies, with ages on the order of 10^6^ generations [13]. An intriguing mystery is why there are huge differences in levels of polymorphism and rates of fixation for inversions between closely related species and between chromosomes within a species, for example in *Drosophila* [4].

## Sex Chromosomes

Inversions have played a key role in the evolution of sex chromosomes. In groups like mammals, the Y chromosome is entirely blocked from recombining with the X chromosome along almost its entire length. Patterns of molecular variation reveal that early in the evolution of the mammalian Y, a series of overlapping inversions progressively extended the size of the nonrecombining portion of the Y [14].

Why should evolution do that? Many genes seem to be under “sex-antagonistic selection,” meaning that alternative alleles are favored in females and males. Theory shows that selection favors decreased recombination between genes under sex-antagonistic selection and the locus that determines sex [15],[16]. It is not difficult to see why: a male-determining chromosome that always carries the allele that enhances male fitness has an advantage over one that sometimes carries the alternative allele that is best in females. Thus, an inversion that captures both the male-determining factor and a male-beneficial allele at another locus will spread. A series of inversions can capture additional loci, binding them into an ever larger nonrecombining block, as apparently happened to the mammalian Y. Once inversions have genetically isolated the Y from the X, the Y evolves as an asexual genetic unit. A series of evolutionary mechanisms then cause the Y to degenerate—these include genetic drift (“Muller's ratchet”) and deleterious mutations that hitchhike to fixation by linkage to advantageous mutations. In the case of groups like mammals and flies, the final result is a Y that is a genetic desert, devoid of almost all of its former genetic content [17].

Inversions may also be critical to the very origin of sex chromosomes. Many (perhaps most) groups of animals and plants that have sex chromosomes do not show the dramatic heteromorphism between X and Y that is familiar from mammals. In the three-spined stickleback, for example, much of sex chromosome recombines and acts otherwise like an autosome. In fact, it was an autosome until recently: sex in its sister species is determined by an entirely different pair of chromosomes [18]. Theory suggests how this might happen: an inversion that captures both a sex-determining mutation and a sex-antagonistic locus on an autosome can form a neo-sex chromosome that can hijack sex determination from the ancestral sex chromosomes [19]. Consistent with this suggestion, the sex determining region of the new Y chromosome in the three-spined stickleback is carried by an inversion. Perhaps the rest of the Y has not yet had time to accumulate inversions down the rest of its length, as happened in mammals.

## Speciation

Inversions are implicated in speciation in several ways. An intriguing pattern is that rates of chromosome evolution and speciation seem to be correlated [20], but that pattern alone does not tell us which factor causes the other, or whether both are driven by a third variable. Some workers, most famously M.J.D. White [21], have argued that fixed inversion differences between species are important for postzygotic isolation because of their underdominant fitness effects. A difficulty with this idea is that drift is unlikely to fix inversions that are strongly underdominant, while those that are more likely to spread because they are only weakly selected will produce little isolation [22]. With favorable demographic conditions (e.g., frequent colonisations and extinction) and life histories (e.g., self-fertilization or close inbreeding), however, models show that populations can fix underdominant chromosomal rearrangements that contribute appreciably to hybrid fitness loss [8].

No matter how fixed differences for inversions between populations get established, we expect them to become hotspots for accumulating positively selected differences and genes that cause incompatibility between species [23]. This is one explanation for an intriguing pattern seen in sunflowers [24] and flies [25]: loci involved in both pre- and postzygotic isolation map to inversions that distinguish closely related species.

An alternative hypothesis is that the inversions in fact became fixed because of adaptive differences that pre-existed at some of those very loci. The idea depends on local adaptation, to which we now turn.

## Local Adaptation

A clear sign that inversions are involved in adaptation comes from geographical variation in their frequency. A dramatic example is the inversion 3RP in *Drosophila melanogaster*, which has established parallel latitudinal clines on three continents [26]. Further, its frequency along the cline has shifted in a way consistent with climatic change over a period of 20 years [27]. Further examples of inversion clines, also correlated with environmental gradients, are seen in several species of *Anopheles*, the mosquito vectors of malaria in Africa [28].

Local adaptation is the situation in which different genes are favored in different environments. An inversion that captures two or more alleles that are adapted to the local environmental conditions has a selective advantage that can cause it to spread [29]. This effect results from suppressed recombination: the new inversion carries only the locally adapted alleles, while the ancestral rearrangement carries mixtures of adapted and maladapted alleles. No epistasis (gene interaction) is needed for the inversion to gain an advantage, which means that this local adaptation mechanism can operate even when the loci are adapting to different environmental variables. This is one reason why the local adaptation mechanism may work much more frequently than other hypotheses that depend, for example, on a delicate balance of genetic interaction to establish inversions.

The local adaptation mechanism can also come into play when two species hybridize: alleles within each species that are adapted to that genetic background experience the same evolutionary forces as genes within a species adapting to different ecological pressures. This proposal can explain why species pairs of *Drosophila* that are sympatric, and thus have the potential to hybridize, differ for inversions more often than allopatric pairs [25]. The theory for the evolution of sex chromosomes sketched above can also be seen as an example of the local adaptation mechanism, with males and females acting as two selective environments. In essence, these hypotheses suggest that inversions spread because they prevent recombination from breaking apart sets of alleles that work well in an ecological or sexual setting. Since recombination continues normally within the populations of inverted and uninverted chromosomes, inversions may escape many of the deleterious consequences suffered by other genetic mechanisms that shut down recombination entirely [30].

## Local Adaptation and Speciation in a Monkeyflower

In this issue of *PLoS Biology*, Lowry and Willis [31] announce the discovery of an inversion in the yellow monkeyflower *Mimulus guttatus* that at once includes some of their most interesting features. This species has a very broad geographical and ecological range in western North America, and occurs in two distinct ecotypes. One is an annual form adapted to the dry habitats commonly found inland. The second is perennial, and is adapted to moist and cool areas typical of the coast. These two forms differ genetically in several ways. Key among the differences is flowering time: the annual form flowers early, before the hot and dry summer, while the perennial form takes advantage of the longer season by investing in more growth and then flowering later. This adaptive difference produces premating isolation, since the two forms are not available at the same time for pollination. It also causes postzygotic isolation, since survival of hybrids is reduced in moist coastal habitats.

Lowry and Willis discovered the inversion by noticing that hybrids between the ecotypes showed no recombination between molecular markers along part of one chromosome. Further, they saw that much of the phenotypic variation that distinguishes the two ecotypes cosegregates with the inversion. The traits involved include three that contribute to reproductive isolation between the forms, as well as other traits such as morphology. The inversion is polymorphic over much of the species' range, and acts as a supergene that makes important contributions to both local adaptation and reproductive isolation between the annual and perennial forms.

This marvelous story seems like a poster child for the local adaptation hypothesis for inversions. A plausible scenario is that an ancestral monkeyflower was under disruptive selection, with the annual form favored in some habitats and the perennial form in others. One day, an inversion arose that captured a set of alleles adapted to one or the other habitat, and it invaded. Currently, other genes throughout the genome also contribute to the differences between the ecotypes. That suggests the possibility that another inversion elsewhere in the genome could appear and invade via the local adaptation mechanism. It is easy to imagine that if this event is repeated one or a few times, what are now two ecotypes within a species will become two distinct species, genetically distinguished by several inversions. Perhaps the yellow monkeyflower is showing us a snapshot of inversions in the process of splitting one species into two.

But this hypothesis has competitors. Perhaps, for example, the breakpoint of the inversion itself has disrupted a gene that has cascading effects on flowering time and growth. Then the inversion evolved not because it prevents recombination between a set of locally adapted genes, but rather as a mutation at a single gene. A second issue is whether the genetic differences that now distinguish the inverted and ancestral chromosomes were responsible for the inversion to invade (as in the local adaptation mechanism), or accumulated after it became established for some other reason.

To test alternative hypotheses for how inversions evolve, we would like to understand what genes or chromosome regions are the targets of selection—are they at the breakpoints, for example, or genes within the inversion? With model organisms, transformations of genes at candidate loci could give strong evidence. But that approach is often not an option, both because the genetic tools are not yet available in most species, and because the effects of transformations generally cannot be tested in natural conditions. An alternative way forward is to use patterns of neutral genetic variation in the DNA inverted and uninverted chromosomes. Under the right conditions, these could be used to find quantitative trait loci (QTL) under divergent selection within the inversion. We might then be able to date the ages of selected alleles relative to the origin of the inversion, to see which came first. Further, different hypotheses, for example selection on the breakpoints versus local adaptation with suppressed recombination, should leave contrasting signatures of neutral genetic variation. Tantalizing hints of such patterns have been seen in in *Drosophila* [32] and *Anopheles* [34]. So far, however, there has been no rigorous test of alternative hypotheses using any approach. A synthesis of genomics and ecological genetics in the style of Lowry and Willis holds the promise of being able to do that before long, with luck before the centenary of Sturtevant's great discovery.
